# HPV Positive Status Is a Favorable Prognostic Factor in Non-Nasopharyngeal Head and Neck Squamous Cell Carcinoma Patients: A Retrospective Study From the Surveillance, Epidemiology, and End Results Database

**DOI:** 10.3389/fonc.2021.688615

**Published:** 2021-09-24

**Authors:** Qiuji Wu, Miao Wang, Yixin Liu, Xulong Wang, Yi Li, Xiaoyan Hu, Ye Qiu, Wenjing Liang, Yongchang Wei, Yahua Zhong

**Affiliations:** ^1^ Department of Radiation and Medical Oncology, Zhongnan Hospital, Wuhan University, Wuhan, China; ^2^ Hubei Key Laboratory of Tumor Biological Behaviors, Zhongnan Hospital, Wuhan University, Wuhan, China; ^3^ Hubei Cancer Clinical Study Center, Zhongnan Hospital, Wuhan University, Wuhan, China

**Keywords:** head and neck squamous cell carcinoma (HNSCC), human papillomavirus (HPV), SEER database, prognosis, nomogram

## Abstract

**Objective:**

To investigate the impact of the human papillomavirus (HPV) status on head and neck squamous cell carcinoma (HNSCC) arising from different anatomic subsites.

**Methods:**

HNSCC patients with known HPV status from the Surveillance, Epidemiology, and End Results (SEER) database between 2010–2015 were included in our analysis. Patients were classified into three categories of HNSCC according to Site recode ICD-O-3/WHO 2008 and Primary Site-labeled, namely, oropharynx, hypopharynx, and nasopharynx. Logistic regression model was conducted to evaluate the relationship between patient characteristics and HPV status. Kaplan-Meier methods and COX regression analysis were used to analyze survival data.

**Results:**

A total of 9,943 HNSCC patients with known HPV status from the SEER database were enrolled, with 6,829 (68.7%) HPV-positive patients. HPV-positive and HPV-negative HNSCC were distinct and had different clinical and socioeconomic features (all P < 0.001). Primary sites, socioeconomical factors (age, sex, marital status, and race), and pathological features (TNM stage and grade) were closely related with HPV status (all P < 0.001). HPV-positive status was a favorable prognostic marker in HNSCC patients with cancers of the oropharynx and hypopharynx (all P < 0.001), but was not in nasopharyngeal carcinoma patients (P = 0.843). A total of 8,933 oropharyngeal carcinoma (OPC) and 558 hypopharyngeal carcinoma (HPC) patients were divided into the training and validation cohorts with a ratio of 1:1. Significant prognostic factors of the OS yielded by multivariate COX analysis in the training cohort were integrated to construct nomograms for OPC and HPC patients. The prognostic models showed a good discrimination with a C-index of 0.79 ± 0.007 and 0.73 ± 0.023 in OPC and HPC, respectively. Favorable calibration was reflected by the calibration curves. Additionally, corresponding risk classification systems for OPC and HPC patients based on the nomograms were built and could perfectly classify patients into low-risk, intermediated-risk, high-risk groups. OS in the three risk groups was accurately differentiated and showed a good discrimination.

**Conclusion:**

HPV positivity was associated with an improved survival in HNSCC patients with cancers of the oropharynx and hypopharynx. Nomograms and corresponding risk classification systems were constructed to assist clinicians in evaluating the survival of OPC and HPC patients.

## Background

Head and neck squamous cell carcinomas (HNSCC) are an atomically heterogeneous group of neoplasms arising from the nasopharynx, oral cavity, oropharynx, hypopharynx, and larynx. Each year, there are approximately 700,000 new cases and 380,000 deaths of HNSCC worldwide (1). It is well known that tobacco smoking, alcohol consumption, and betel quid chewing in Iran and some Southeast Asian countries are classical etiological factors for HNSCC development (2, 3). Virus infection is another important etiological cause of HNSCC. For instance, Epstein-Barr virus (EBV) is common and strongly associated with nasopharyngeal carcinoma (NPC) in Southern China and Southeast Asian countries (4). And it has become clear that high-risk human papillomavirus (HPV) infection is an important etiological and prognostic factor for a subset of HNSCC over the last decade (5–7). Moreover, there is a solid epidemiological work showing that HPV-related HNSCC is on the rise in the Western world with an increased incidence of HPV infection in HNSCC of approximately 50%, while a decrease in incidence of smoking-related HNSCC is seen due to an effective smoking control (8, 9).

In fact, HPV infection as an established cause and a risk stratification biomarker in oropharyngeal carcinoma (OPC) is well known (10). Guidelines have recommended that all OPC patients should be tested for HPV status and HPV-positive OPC was specified separately as an independent entity in the eighth edition of the American Joint Committee on Cancer (AJCC) tumor node metastasis (TNM) staging system (11–13). OPC patients with HPV positivity showed an improved response to therapy and a better survival (14, 15). However, the role of HPV infection in non-oropharyngeal HNSCC of the nasopharynx, oral cavity, and hypopharynx was not well defined despite HPV infection being present in 7% to 25% of non-oropharyngeal HNSCC. Results in some published studies about this topic were inconsistent. For example, several retrospective studies showed that HPV positivity was associated with an improved survival in patients with HNSCC from the oral cavity, hypopharynx, and nasopharynx (16–19). While some studies reported that there was no survival difference among HPV-positive and HPV-negative non-oropharyngeal patients (20–22), even a detrimental role of HPV positivity in HPV-positive ones (23–25). Large sample research to explore the role of HPV status in non-oropharyngeal patients was warranted. And to the best of our knowledge, there is no prognostic model including the HPV status for HNSCC patients.

Therefore, in this study, we sought to investigate the prognostic role of HPV status in HNSCC from different subsites based on the data from the Surveillance, Epidemiology, and End Results (SEER) database. We further established an HPV-based nomograms to predict the survival probability and provided a risk classification tool for OPC and hypopharyngeal squamous cell carcinoma (HPC) patients.

## Patients and Methods

### Cohort Population

We performed a retrospective research based on information from the SEER database, a publicly available cancer statistics database, which is constitutive of 18 cancer registries in the United States and covers about 28% of the total population of the United States (https://seer.cancer.gov/data/). Informed consent was waived for the use of public data from the SEER.

A customized Surveillance, Epidemiology, and End Results (SEER) Head and Neck with HPV Status Database was used to collect adult patients (>18 years old) who were diagnosed as first primary HNSCC with known HPV status from 2010 to 2015 in our study. We verified the specific sites of the primary cancers of each patient according to Site recode ICD-O-3/WHO 2008 and Primary Site-labeled, and classified included patients into three categories of HNSCC: oropharynx (C01.9, C02.4, C05.1, C05.2 C09.0, C09.1, C09.8, C09.9, C10.0, C10.2, C10.3, C10.4, C10.8, C10.9), hypopharynx (C12.9, C13.0, C13.1, C13.2, C13.8, C13.9), nasopharynx(C11.0, C11.1, C11.2, C11.3, C11.8, C11.9), which was similar to the classification criteria in terms of the anatomical subsites in a previous study based on the National Cancer Data Base (26). All included cases were diagnosed as squamous cell carcinoma by positive histology confirmation with the ICD-O-3 histologic type codes 8052, 8053, 8070–8076, 8078, 8083, 8084, and 8094. TNM staging in the SEER database between 2010–2015 was recorded according to AJCC seventh edition. Patients with unknown survival data and TNM stage, and more than one primary tumor were excluded.

Data of race, age at diagnosis, gender, marital status, primary sites, pathology grade, HPV status, treatment (primary surgery, radiation, and chemotherapy), and survival time were collected. The endpoint for the current study was the overall survival (OS), which was defined as the time from cancer diagnosis to the time of death from any cause or of the last follow-up.

### Statistical Analysis

Descriptive statistics were used to compare the demographic and clinical characteristics of patients between HPV-positive and HPV-negative HNSCC patients. Categorized variables, presented as frequency and their proportion, were analyzed by Chi-square tests. Logistic regression analysis was applied to analyze the associations between the clinicopathologic factors and tumor HPV status. Kaplan-Meier analyses were performed to generate survival curves and Log Rank test was applied to compare the differences among the curves. Comparative risk factors of the overall survival (OS) were identified by univariate and multivariate analysis using Cox regression models. Simple random sampling was performed with the random sampling function [sample () function] in the R software, and a total of 8,933 OPC and 558 HPC patients were randomly classified into the training and validation groups by a ratio of 1 to 1. The data of the training cohort was used to establish the nomogram at the 3- and 5-year OS with the “rms” package. Concordance index (C-index) and area under curve (AUC) were calculated to evaluate the discrimination of the established nomograms. Calibration plots were used to evaluate the calibrating ability. C-index and AUC values vary from 0.5 to 1.0, where 0.5 represents random chance and 1.0 indicates a perfect fit. Typically, C-index and AUC values greater than 0.7 suggest a reasonable estimation. All statistical analyses were conducted using the statistical software packages R version 3.6.2 (http://www.R-project.org, The R Foundation) and SPSS statistics version 23.0 (IBM SPSS Statistics, New York, United States). All statistical tests were two-sided and a P-value < 0.05 was considered statistically significant.

## Results

### Patient Characteristics Among HPV-Positive and HPV-Negative Head and Neck Squamous Cell Carcinoma Patients

Overall, a total of 9,943 HNSCC patients with known HPV status from the SEER database were enrolled in this study, including 6,829 (68.7%) HPV-positive patients and 3,114 (31.3%) HPV-negative patients. As shown in **Table 1**, significant clinical and socioeconomic differences were observed among the HPV-positive and HPV-negative groups. HPV-positive HNSCC is more common in younger patients (50–69 years old: 73.4% *vs*. 66.5% for HPV-positive *vs* HPV-negative) and in married patients (62.0% *vs*. 50.0%). Regarding the gender of patients, HPV-positive *versus* HPV-negative was 89.3% *vs*. 70.9% in male patients, while it was 23.4% *vs*. 13.3% in female patients (P < 0.001). In terms of ethnic difference, HPV-positive *versus* HPV-negative was 90.2% *vs*. 77.5% in white patients, while it was 5.4% *vs*. 13.5% in black patients (P < 0.001). Compared to HPV-negative HNSCC, HPV-positive HNSCC were more likely to occur in the oropharynx (95.6% *vs*. 77.2%) subsites but occurred less in the nasopharynx (2.3% *vs*. 9.4%) and hypopharynx (2.1% *vs*. 13.4%) subsites (P < 0.001 for all). HPV-positive patients presented with a lower ratio of bulky primary tumor (T3: 17.4% *vs*. 22.3%; T4: 14.2% *vs*. 23.4%, P < 0.001) and distant metastasis (M1: 2.8% *vs*. 5.0%, P < 0.001) but a higher rate of N2 stage disease (63.6% *vs*. 50.8%, P < 0.001). As a result, HPV-positive patients had more stage IVA–B diseases (69.7% *vs*. 59.4%, P < 0.001). Besides, a higher ratio of grade III–IV disease was seen in HPV-positive patients (48.2% *vs*. 38.5%, P < 0.001). As for treatment, HPV-positive HNSCC patients underwent more anti-tumor therapies including surgery (39.6% *vs*. 29.7%, P < 0.001), radiotherapy (89.6% *vs*. 83.9%, P < 0.001), and chemotherapy (74.6% *vs*. 71.7%, P < 0.001).

**Table 1:** Clinical and demographic features of HNSCC population according to HPV status.

Characteristics	Total	HPV (+)	HPV (-)	P-value
	N = 9,943 (100%)	N = 6,829 (68.7%)	N = 3,114 (31.3%)	
**Primary site**				**<0.001**
Nasopharynx	452 (4.5%)	159 (2.3%)	293 (9.4%)	
Hypopharynx	558 (5.6%)	140 (2.1%)	418 (13.4%)	
Oropharynx	8,933 (89.8%)	6,530 (95.6%)	2,403 (77.2%)	
**Age (years)**				**<0.001**
18–49	1,326 (13.3%)	916 (13.4%)	410 (13.2)	
50–69	7,085 (71.3%)	5,015 (73.4%)	2,070 (66.5)	
>=70	1,532 (15.4%)	898 (13.1%)	634 (20.4)	
**Race**				**<0.001**
Black	792 (8.0%)	372 (5.4%)	420 (13.5)	
White	8,577 (86.3%)	6,163 (90.2%)	2,414 (77.5)	
Other^#^	536 (5.4%)	266 (3.9%)	270 (8.7%)	
Unknown	38 (0.4%)	28 (0.4%)	10 (0.3%)	
**Gender**				**<0.001**
Male	8,308 (83.6%)	6,100 (89.3%)	2,208 (70.9%)	
Female	1,635 (16.4%)	729 (13.3%)	906 (23.4%)	
**Marital status**				**<0.001**
Married	5,794 (58.3%)	4,236 (62.0%)	1,558 (50.0%)	
Non-married	3,702 (37.2%)	2,288 (33.5%)	1,414 (45.4%)	
Unknown	447 (4.5%)	305 (4.5%)	142 (4.6%)	
**Grade**				**<0.001**
Grade I–II	3,312 (33.3%)	1,989 (29.1%)	1,323 (42.5%)	
Grade III–IV	4,492 (45.2%)	3,293 (48.2%)	1,199 (38.5%)	
Unknown	2,139 (21.5%)	1,547 (22.7%)	592 (19.0%)	
**AJCC 7^th^ stage**				**<0.001**
Stage I	388 (3.9%)	189 (2.8%)	199 (6.4%)	
Stage II	693 (7.0%)	418 (6.1%)	275 (8.8%)	
Stage III	1,907 (19.2%)	1,271 (18.6%)	636 (20.4%)	
Stage IVa–b	6,611 (66.4%)	4,951 (69.7%)	1,848 (59.4%)	
Stage IVc	344 (3.5%)	188 (2.8%)	156 (5.0%)	
**Tumor stage**				**<0.001**
T1	2,624 (26.4%)	1,934 (28.3%)	690 (22.2%)	
T2	3,738 (37.6%)	2,738 (40.1%)	1,000 (32.1%)	
T3	1,882 (18.9%)	1,187 (17.4%)	695 (22.3%)	
T4	1,699 (17.1%)	970 (14.2%)	729 (23.4%)	
**Nodal stage**				**<0.001**
N0	1,619 (16.3%)	917 (13.4%)	702 (22.5%)	
N1	1,878 (18.9%)	1,237 (18.1%)	641 (20.6%)	
N2	5,925 (59.6%)	4,343 (63.6%)	1,582 (50.8%)	
N3	521 (5.2%)	332 (4.9%)	189 (6.1%)	
**Metastatic stage**				**<0.001**
M0	9,599 (96.5%)	6,641 (97.2%)	2,958 (95.0%)	
M1	344 (3.5%)	188 (2.8%)	156 (5.0%)	
Unknown				**<0.001**
**Surgery for primary site**				
No	6,303 (63.4%)	4,118 (60.3%)	2,185 (70.2%)	
Yes	3,631 (36.5%)	2,705 (39.6%)	926 (29.7%)	
Unknown	9 (0.1%)	6 (0.1%)	3 (0.1%)	
**Radiotherapy**				**<0.001**
No	1,213 (12.2%)	713 (10.4%)	500 (16.1%)	
Yes	8,730 (87.8%)	6,116 (89.6%)	2,614 (83.9%)	
**Chemotherapy**				**0.003**
No	2,613 (26.3%)	1,733 (25.4%)	880 (28.3%)	
Yes	7,330 (73.7%)	5,096 (74.6%)	2,234 (71.7%)	

^#^American Indian/AK Native, Asian/Pacific Islander.

HPV, human papillomavirus; HNSCC, head and neck squamous cell carcinomas; AJCC, American Joint Committee on Cancer.

The bold values indicated that P-value was less than 0.05 and the difference was statistically significant.

### The Association Between Patient Characteristics and Tumor Human Papillomavirus Status

**Figure 1:**
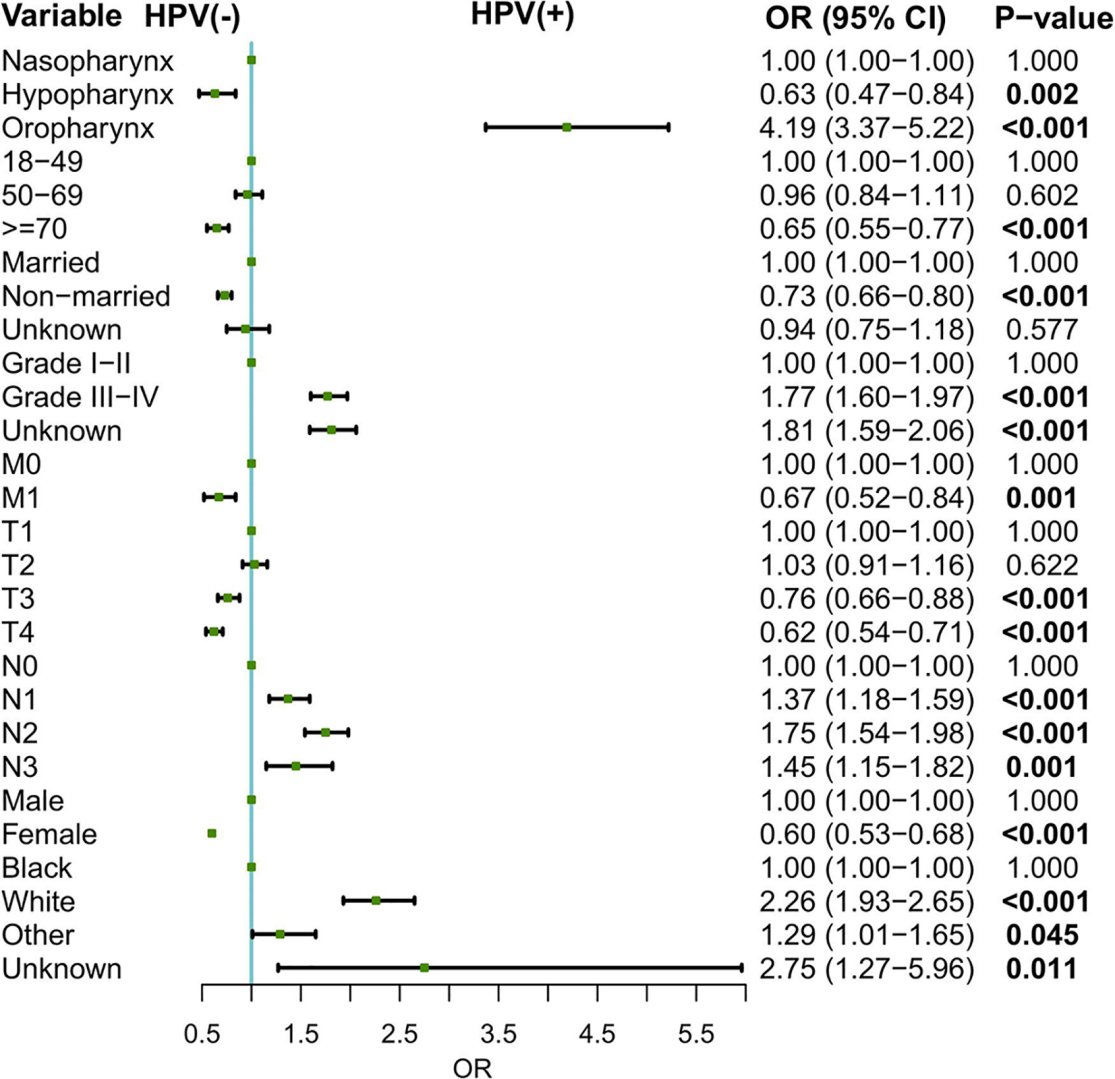
Associations between patient characteristics and HPV status. HPV, human papillomavirus.

Logistic regression model was conducted to evaluate the relationship between patient characteristics and tumor HPV status. As shown in **Figure 1**, oropharynx as the primary sites (oropharynx: OR = 4.19, 95% CI: 3.37–5.22, compared to the nasopharynx), grade III–IV disease (OR = 1.77, 95% CI: 1.60–1.97, for grade III–IV *vs*. grade I–II), nodal involvement (N1: OR = 1.37, 95% CI: 1.18–1.59, N2: OR = 1.75, 95% CI: 1.54–1.98, N3: OR = 1.45, 95% CI: 1.15–1.82, compared to N0), and white race (OR = 2.26, 95% CI: 1.94–2.63, for white *vs*. black) were associated with higher odds of an HPV-positive status. In contrast, hypopharynx as the primary sites (OR = 0.63, 95% CI: 0.47–0.84, for hypopharynx *vs*. nasopharynx), female (OR = 0.60,95% CI: 0.53–0.68, for female *vs*. male), older age at diagnosis (OR = 0.65, 95% CI: 0.55–0.77 for >=70 *vs*. 18–49), non-married status (OR = 0.73, 95% CI: 0.66–0.80, for non-married *vs*. married), and distant metastasis (OR = 0.67, 95% CI: 0.52–0.84, for M1 *vs*. M0) might be associated with the decreased odds of an HPV-positive HNSCC.

### Survival Analysis and Prognostic Factors

**Figure 2:**
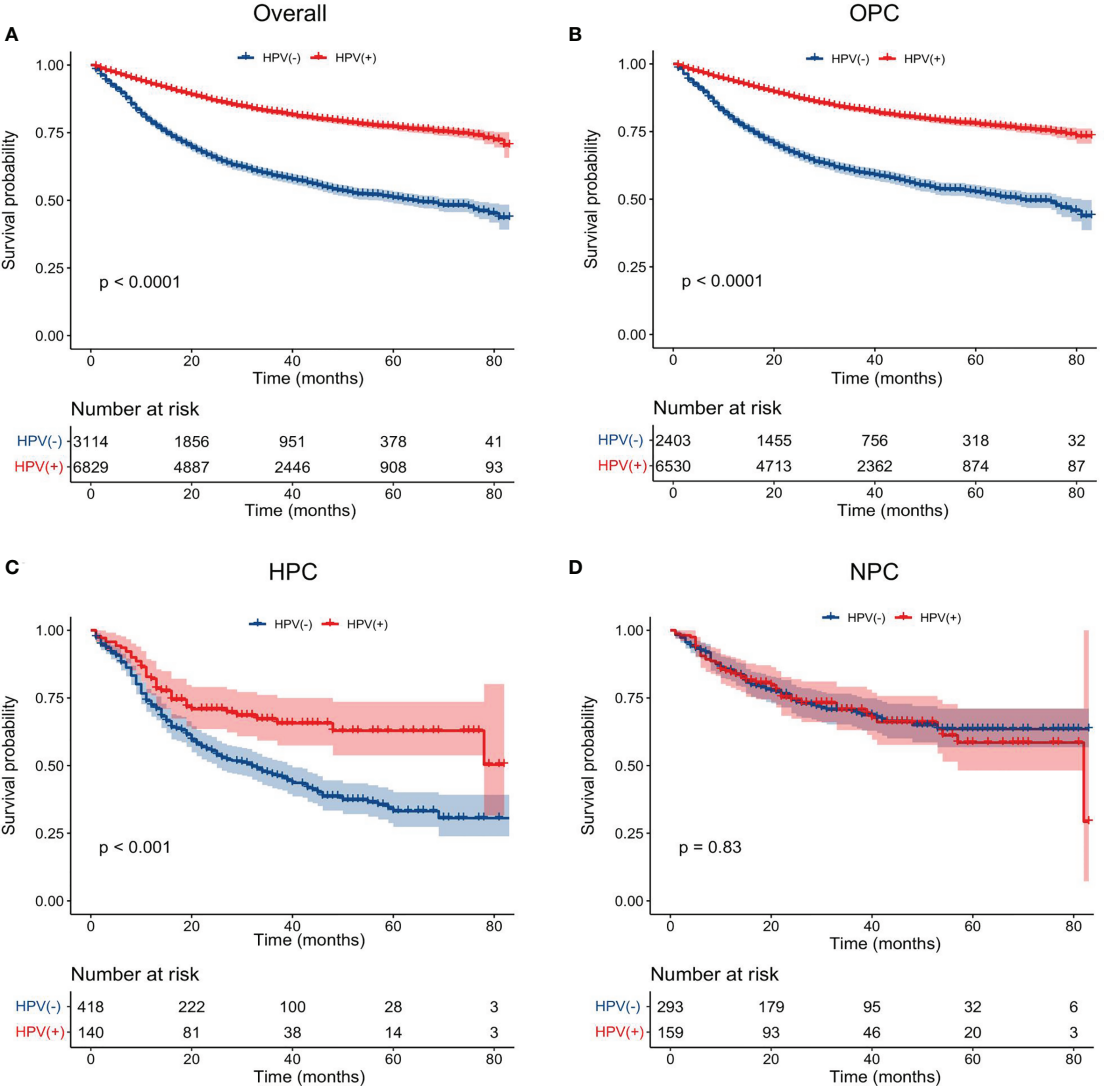
Impact of HPV infection on the overall survival of HNSCC patients arising from different anatomical subsites. K-M plots of the overall survival were shown for: **(A)** total population, **(B)** oropharyngeal carcinoma, **(C)** hypopharyngeal carcinoma, **(D)** nasopharyngeal carcinoma. HPV, human papillomavirus; HNSCC, head and neck squamous cell carcinoma; OPC, oropharyngeal carcinoma; HPC, hypopharyngeal carcinoma; NPC, nasopharyngeal carcinoma.

The median follow-up period for the entire study population was 37 months (95% CI: 36.30–37.70 months). Kaplan–Meier estimates demonstrated that HPV-positive HNSCC patients had a better survival than that of HPV-negative HNSCC patients (P < 0.001) (**Figure 2A**). The estimated 3- and 5-year OS rates were 80.0% and 75.0%, respectively, for HPV-positive HNSCC patients, compared with 54.0% and 48.0% for HPV-negative patients. Multivariate Cox regression analysis showed that HPV status was an independent prognostic factor for the OS in the overall HNSCC population (**Supplementary Table 1**). Compared to HPV-negative patients, patients with HPV-positive HNSCC had an improved OS (HR = 0.51, 95% CI: 0.46–0.55, P < 0.001). Other factors associated with the OS in the multivariate analysis included primary site, age, race, T stage, N stage, M stage, grade, marital status, primary site surgery, radiotherapy, and chemotherapy.

Next, to clarify the prognostic effect of HPV infection on different HNSCC, we compared the survival data of the OS between HPV-positive and HPV-negative HNSCC patients of different anatomical subsites (oropharynx, hypopharynx, and nasopharynx). The survival curves intuitively illustrated that, in the oropharynx and hypopharynx, patients with HPV-positive cancers showed a better OS than that of their counterparts with HPV-negative cancers (P < 0.001) (**Figures 2B, C**). While for nasopharynx cancers, HPV-positive patients had a similar OS to that of HPV-negative patients (P = 0.83) (**Figure 2D**). After adjusting for age, marital status, race, gender, T stage, N stage, M stage, primary site surgery, chemotherapy, and radiation, we found that an HPV-positive status was associated with an improved OS in oropharynx (HR = 0.48, 95% CI: 0.44–0.53, P < 0.001, for HPV-positive *vs*. HPV-negative) and hypopharynx locations (HR = 0.60, 95% CI: 0.43–0.84, P = 0.002, for HPV-positive *vs*. HPV-negative) (**Table 2**). However, there is no significant association between the HPV status and OS in NPC patients (HR = 1.04, 95% CI: 0.71–1.52, P = 0.843) after adjusting for these potential possible factors.

**Table 2:** Univariate and multivariate analyses of the overall survival in HNSCC arising from the oropharynx, hypopharynx, and nasopharynx.

HPV (+) *vs* HPV (-) in subsites	Univariate analyses		Multivariate analyses^#^	
	HR (95% CI)	P-value	HR (95% CI)	P-value
**Oropharynx [HPV (+) *vs*. HPV (-)]**	0.36 (0.33–0.39)	**<0.001**	0.48 (0.44–0.53)	**<0.001**
**Hypopharynx [HPV (+) *vs*. HPV (-)]**	0.54 (0.39–0.74)	**<0.001**	0.60 (0.43–0.84)	**0.002**
**Nasopharynx [HPV (+) *vs*. HPV (-)]**	1.04 (0.73–1.49)	0.826	1.04 (0.71–1.52)	0.843

**^#^** Adjusted for age, marital status, race, gender, T stage, N stage, M stage, surgery, chemotherapy, and radiation in multivariate analyses.

HR, Hazard Ratio; 95% CI, 95% confidence interval; OS, overall survival; HNSCC, head and neck squamous cell carcinoma.

The bold values indicated that P-value was less than 0.05 and the difference was statistically significant.

### Construction and Validation of the Nomograms

**Figure 3:**
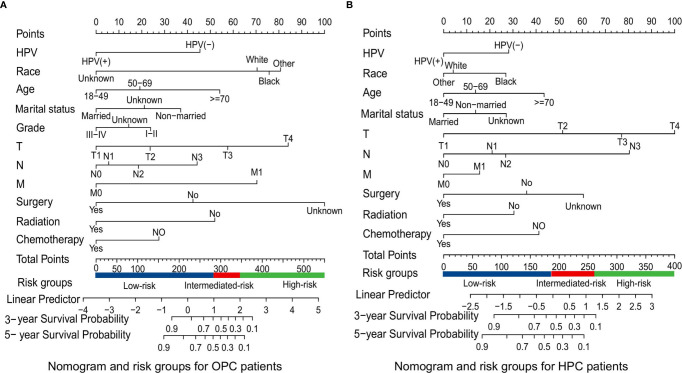
Survival nomograms and risk groups for OPC and HPC patients. **(A)** Prediction of the 3- and 5-year OS in OPC patients and the risk groups based on the total points of each OPC patient in the training cohort; **(B)** Prediction of the 3- and 5-year OS in HPC patients and the risk groups based on the total points of each HPC patient in the training cohort. OS, overall survival; OPC, oropharyngeal carcinoma; HPC, hypopharyngeal carcinoma.

**Figure 4:**
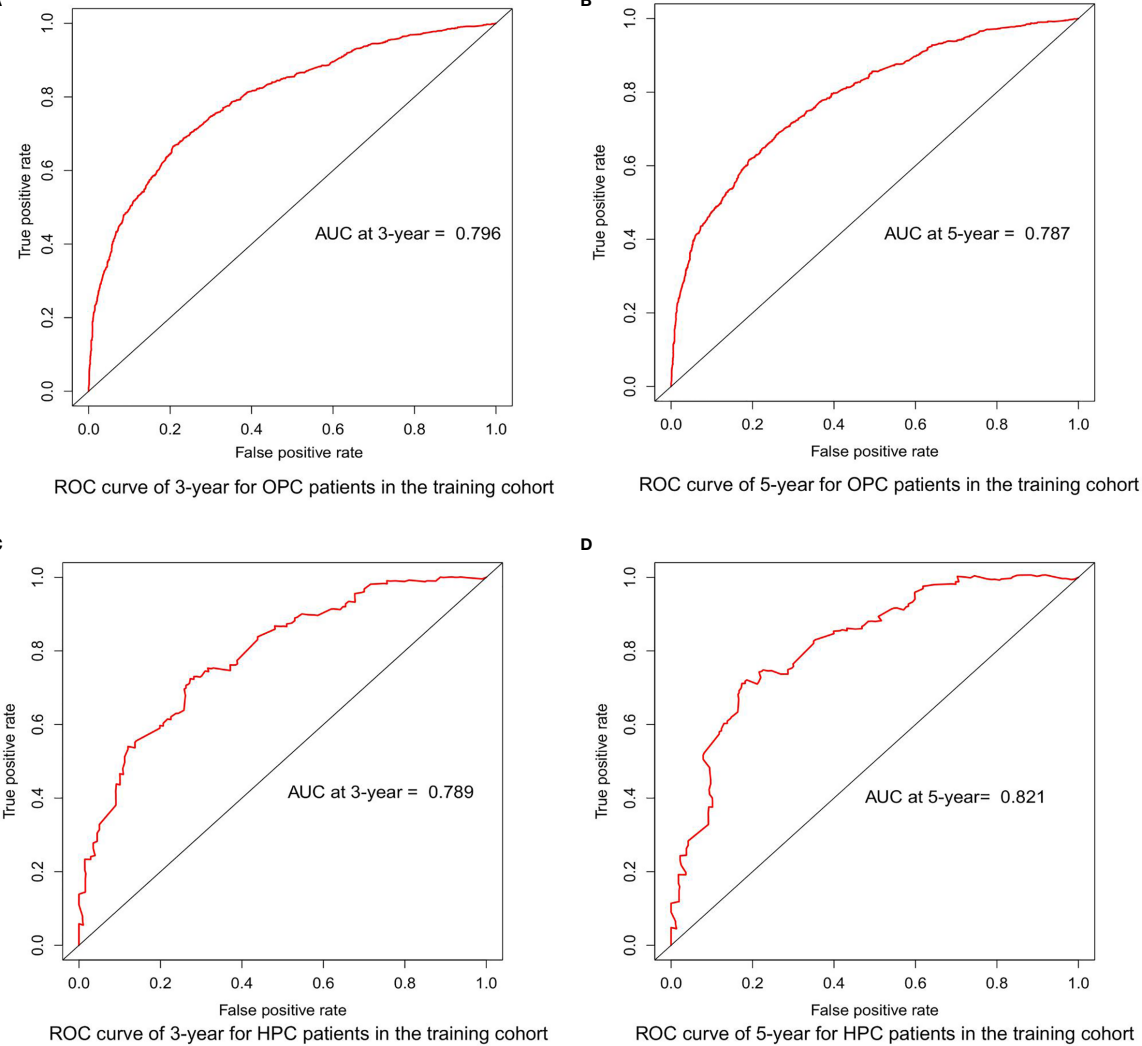
ROC curves depicting the predictive performance of the survival nomograms in the training cohorts. **(A, B)** ROC curves for the 3- and 5-year OS of OPC patients in the training cohort; **(C, D)** ROC curves for the 3- and 5-year OS of HPC patients in the training cohort. ROC, receiver-operating characteristic; OS, overall survival; FP, false positive; TP, true positive; OPC, oropharyngeal carcinoma; HPC, hypopharyngeal carcinoma.

**Figure 5:**
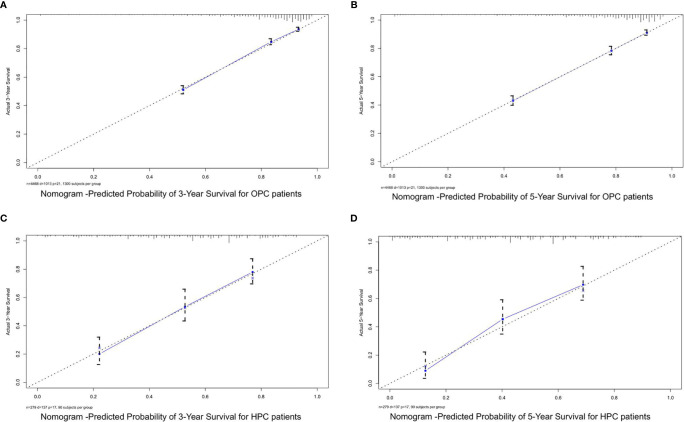
The calibration curves for predicting the OS of OPC and HPC patients in the training cohorts. **(A, B)** Calibration curves for the 3- and 5-year OS of OPC patients in the training cohort; **(C, D)** calibration curves for the 3- and 5-year OS of HPC patients in the training cohort. OS, overall survival; OPC, oropharyngeal carcinoma; HPC, hypopharyngeal carcinoma.

Based on the mentioned results and the fact that HPV infection was significantly associated with the prognosis of OPC and HPC patients, we respectively established and validated the prognostic models for OPC and HPC patients. First, we randomly classified a total of 8,933 OPC patients and 558 HPC patients with known HPV status into the training and validation groups by a ratio of 1 to 1. Baseline clinical characteristics of OPC and HPC patients among the training and validation groups are listed in **Supplementary Table 2** and no statistical intra-group difference was observed. Univariate and multivariate Cox regression analysis of training sets were performed to identify significant prognostic factors of the OS for both OPC and HPC patients (**Table 3**). All statistically and clinically significant prognostic indicators for the OS were integrated to construct the prognostic models. Prognostic models for OPC and HPC patients were virtually presented in the form of a nomogram (**Figures 3A, B**) and were validated using the validation cohort. The nomogram for OPC was constructed based on 11 important prognostic factors including HPV status, race, age, marital status, grade, T stage, N stage, M stage, primary site surgery, chemotherapy, and radiotherapy, and 10 statistically and clinically significant prognostic indicators (HPV status, race, age, marital status, T stage, N stage, M stage, primary site surgery, chemotherapy, and radiotherapy) were integrated to establish the nomogram for HPC patients. The two models showed a good discrimination, with the C-index for the prediction of the OS respectively being 0.79 ± 0.007 and 0.73 ± 0.023 for OPC and HPC. The AUC values at 3- and 5-year in OPC were 0.796 and 0.787 (**Figures 4A, B**) and they were 0.789 and 0.821 in HPC (**Figures 4C, D**), respectively, indicating that the established nomograms exhibited a good predictive performance. The calibration curves showed a good calibration with an optimal agreement between the predicted nomograms and actual OS at 3 years and 5 years (**Figure 5**). Application of the nomogram for OPC in the validation cohort still gave a good discrimination and good calibration as shown in **Supplementary Figures 1A, B** and **Supplementary Figures 2A, B**. Similar results were observed for HPC patients in the validation cohort (**Supplementary Figures 1C, D** and **Supplementary Figures 2C, D**).

**Table 3:** Univariate and multivariate cox analysis of the overall survival for patients with oropharyngeal carcinoma (OPC) and Hypopharyngeal carcinoma (HPC) in the training groups.

Covariate	OPC				HPC			
	Univariate analysis		Multivariable analysis		Univariate analysis		Multivariable analysis	
	HR (95% CI)	P-value	HR (95% CI)	P-value	HR (95% CI)	P-value	HR (95% CI)	P-value
**HPV status**								
HPV (-)	–	–	–	–	–	–	–	–
HPV (+)	0.36 (0.32–0.41)	**<0.001**	0.49 (0.43–0.56)	**<0.001**	0.51 (0.32–0.79)	0.003	0.61 (0.38–0.97)	**0.038**
**Age**								
18–49	–	–	–	–	–	–	–	–
50–69	1.47 (1.18–1.85)	**0.001**	1.35 (1.08–1.70)	**0.010**	1.21 (0.58–2.49)	0.612	1.27 (0.61–2.67)	0.524
>=70	3.12 (2.44–3.98)	**<0.001**	2.33 (1.81–3.00)	0.218	1.80 (0.85–3.84)	**0.126**	2.13 (0.97–4.70)	0.060
**Race**								
Black	–	–	–	–	–	–	–	–
White	0.47 (0.39–0.57)	**<0.001**	0.92 (0.76–1.11)	0.395	0.55 (0.37–0.83)	**0.005**	0.67 (0.43–1.06)	0.087
Other^#^	0.63 (0.44–0.89)	**0.009**	1.08 (0.75–1.54)	0.678	0.67 (0.34–1.34)	0.259	0.62 (0.29–1.33)	0.222
Unknown	0.15 (0.02–1.05)	0.056	0.29 (0.04–2.08)	**<0.001**				
**Gender**								
Male	–	–	–	–	–	–	–	–
Female	1.40 (1.20–1.64)	**<0.001**	1.13 (0.96–1.33)	0.136	1.26 (0.83–1.89)	0.278	–	–
**Marital status**								
Married	–	–	–	–	–	–	–	–
Non-married	2.33 (2.05–2.65)	**<0.001**	1.78 (1.55–2.03)	**<0.001**	1.56 (1.10–2.22)	**0.014**	1.27 (0.85–1.90)	0.242
Unknown	2.33 (2.05–2.65)	**0.001**	1.38 (1.04–1.85)	**0.028**	1.63 (0.87–3.05)	0.126	1.60 (0.83–3.10)	0.161
**Grade**								
Grade I–II	–	–	–	–	–	–	–	–
Grade III–IV	0.60 (0.53–0.70)	**<0.001**	0.68 (0.59–0.79)	**<0.001**	0.89 (0.61–1.29)	0.537	–	–
Unknown	0.86 (0.73–1.01)	0.059	0.86 (0.73–1.02)	0.080	0.97 (0.62–1.53)	0.900	–	–
**T stage**								
T1	–	–	–	–	–	–	–	–
T2	1.51 (1.24–1.85)	**<0.001**	1.47 (1.20–1.81)	**<0.001**	2.60 (1.02–6.61)	0.045	2.46 (0.94–6.44)	0.067
T3	3.12 (2.53–3.84)	**<0.001**	2.53 (2.03–3.15)	**<0.001**	3.48 (1.38–8.83)	**0.008**	3.84 (1.47–10.00)	**0.006**
T4	5.67 (4.66–6.90)	**<0.001**	3.85 (3.11–4.75)	**<0.001**	5.59 (2.23–14.02)	**<0.001**	5.75 (2.22–14.85)	**<0.001**
**N stage**								
N0	–	–	–	–	–	–	–	–
N1	0.87 (0.70–1.08)	0.212	1.10 (0.88–1.38)	0.403	1.15 (0.69–1.93)	0.595	1.44 (0.83–2.50)	0.192
N2	0.97 (0.82–1.16)	0.779	1.36 (1.13–1.64)	**0.001**	1.3 (0.86–1.98)	0.219	1.60 (1.00–2.55)	**0.050**
N3	2.05 (1.57–2.68)	**<0.001**	2.05 (1.54–2.72)	**<0.001**	2.51 (1.19–5.28)	**0.015**	4.06 (1.80–9.15)	**0.001**
**M stage**								
M0	–	–	–	–	–	–	–	–
M1	5.63 (4.58–6.93)	**<0.001**	3.06 (2.45–3.82)	**<0.001**	2.57 (1.47–4.49)	**0.001**	1.31 (0.70–2.47)	0.4025
**Surgery for primary site**								
No	–	–	–	–	–	–	–	–
Yes	0.39 (0.33–0.45)		0.51 (0.43–0.6)		0.58 (0.36–0.94)	**0.026**	0.53 (0.32–0.89)	**0.016**
Unknown	1.38 (0.19–9.78)	0.749	2.37 (0.33–17.11)	0.394	–	–	–	–
**Radiotherapy**								
No	–	–	–	–	–	–	–	–
Yes	0.41 (0.35–0.48)	**<0.001**	0.44 (0.37–0.52)	**<0.001**	0.50 (0.32–0.76)	**0.002**	0.59 (0.35–1.00)	**0.048**
**Chemotherapy**								
No	–	–	–	–	–	–	–	–
Yes	0.76 (0.66–0.87)	**<0.001**	0.65 (0.55–0.76)	**<0.001**	0.59 (0.40–0.86)	**0.006**	0.49 (0.30–0.79)	**0.004**

^#^American Indian/AK Native, Asian/Pacific Islander.

HPV, human papillomavirus; HNSCC, head and neck squamous cell carcinomas; AJCC, American Joint Committee on Cancer.

The bold values indicated that P-value was less than 0.05 and the difference was statistically significant.

### Risk Classification System

**Figure 6:**
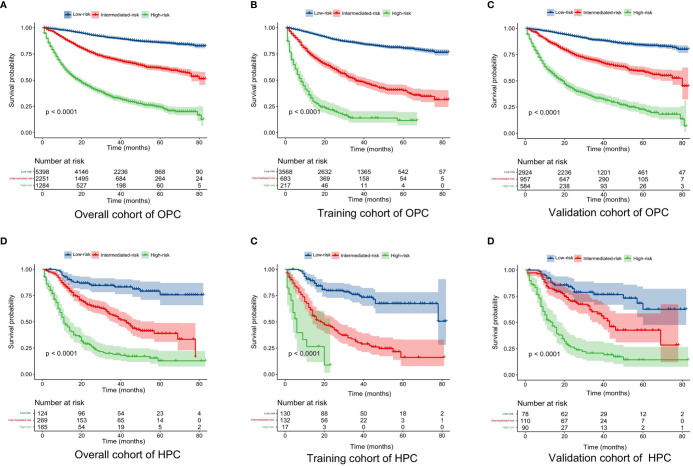
Kaplan–Meier curves of the OS for OPC and HPC patients in the low-, intermediate-, and high-risk groups. **(A–C)**: Kaplan–Meier curves of the OS for OPC patients in the overall, training, and validation cohort; **(D–F)** Kaplan–Meier curves of the OS for HPC patients in the overall, training, and validation cohort. OS, overall survival; OPC, oropharyngeal carcinoma; HPC, hypopharyngeal carcinoma.

Additionally, the corresponding risk classification systems of the OS for OPC and HPC patients were constructed, according to the cutoff analyses for the total points of each patient in the total cohort by the X-title program. All OPC patients were classified into three risk groups: the low-risk (score ≤ 272), intermediate-risk (score 272–345), and high-risk (score ≥ 345) groups. Similarly, all HPC patients were divided into the low-risk group (score ≤ 188), intermediate-risk group (score 188–264), and high-risk group (score ≥ 264). Further Kaplan–Meier curves showed that there was a remarkable survival difference among the three risk groups in both OPC (**Figures 6A–C**) and HPC (**Figures 6D–F**) patients.

## Discussion

In the present study, we aimed to determine the role of tumor HPV status in HNSCC from different subsites (oropharynx, nasopharynx, and hypopharynx), based on the customized SEER Head and Neck with HPV Status Database. We found that HPV positivity was related to a superior survival in OPC and HPC patients, but not in patients with NPC. We also established nomograms including the HPV status that predicted the 3- and 5-year overall survival for OPC and HPC patients.

In this database, HPV infection was prevalent (68.7%) among HNSCC patients, even in non-oropharyngeal HNSCC, the HPV-positivity in cancers of the oropharynx, hypopharynx, and nasopharynx were 73.1% (6530/8933), 25.1% (140/558), 35.2% (159/452), respectively. This prevalence was similar to what has been reported in the Western population (27). Notably, all patients in this database were detected for HPV infection in their tumors, while those who did not have HPV infection detection information were not included. This would inevitably lead to a selection bias. Another factor that might further add to the information bias was the fact that we could not distinguish the exact detection methods used to determine the HPV infection status and genotypes of HPV. Direct and specific test of HPV such as HPV DNA and RNA detection were sensitive but more complicated and more expensive (28). On the other hand, immunohistochemical staining of the p16 protein was widely used as a surrogate marker of HPV infection and has been recommended by the Eighth edition of the TNM Classification system in oropharyngeal carcinoma (29, 30). A large international study of 3,680 samples showed that HPV infection in OPC, oral cavity carcinoma, and larynx carcinoma were 22.4%, 4.4%, and 3.5%, respectively, based on positivity for HPV-DNA, and for either HPV E6 mRNA or p16, and were 18.5%, 3.0%, and 1.5%, respectively, when requiring a simultaneous positivity for all three markers (31). Another study found that there might be a considerable (up to 26.2%) misclassification when using p16 staining alone to determine the HPV infection status (32). It would be ideal if there was a standard HPV detection method that stratify patient outcomes well while being clinically practical and inexpensive. Nevertheless, with the current information we could obtain from the SEER head and neck cancer with HPV status database, we could still find convincing clues about how HPV infection would impact the survival of a head and neck cancer patient.

In the present study, we mainly focus on the prognostic role of HPV status in HNSCC from different subsites (oropharynx, nasopharynx, and hypopharynx). We found that HPV status was not only an important prognostic marker in patients with OPC but also an important prognostic factor in patients with HNSCC from the hypopharynx. In other words, HPV status was significantly associated with the prognosis of non-nasopharyngeal HNSCC. Based on the fact of that an HPV-positive status was a crucial prognostic factor, we developed and validated prognostic nomograms that integrated the HPV status for OPC and HPC patients, respectively. Our established nomograms for OPC and HPC patients performed well in calibration and discrimination, showing a good predictive value. Moreover, based on the total points produced by the nomograms, we developed a novel risk classification system for OPC and HPC patients, which classified patients into low-, intermediate-, and high-risk groups. Significant difference in the OS was observed among the three prognostic groups in the three cohorts. Therefore, by using the nomograms, we could accurately predict the individual survival probability at a certain timepoint and make a risk classification for OPC and HPC patients. But the present nomograms were established and validated by using the data from the same database, thus, a prospective validation of the nomograms in another independent dataset is warranted for a reliable evaluation.

HPV infection was not a clinically prognostic marker for NPC patients in our study. Previous studies had showed that HPV infection in NPC was observed but it was relatively rare compared to EBV infection and the prognostic role of HPV infection in NPC was controversial (33). There were studies suggesting that there was no statistical difference in the survival between HPV positive and EBV positive NPC patients (34). While, existing literature showed that HPV positive patients had worse outcomes compared to patients with EBV-positive NPC (35). It was well known that NPC was strongly associated with EBV infection and plasma EBV DNA have been used for population screening, prognostication, predicting treatment response for therapeutic adaptation, and disease surveillance in NPC (36, 37). However, the role of HPV in NPC or its interplay with EBV was unclear despite of an increased awareness of HPV infection in NPC. In the current study, we could not further evaluate the role of EBV and its interplay with HPV as the EBV information in the SEER database was unavailable. Therefore, future studies exploring the role of HPV in NPC or its interplay with EBV are needed.

In addition, we explored the association between patient characteristics and HPV status. The results showed that primary sites, socioeconomical factors, and pathological features are closely with the HPV status. Specifically, patients who were married, at younger age, male, and white race were more likely to present with an HPV-positive HNSCC. The results were consistent with previous literatures (38, 39). This might imply that patients with these characteristics were more vulnerable to HPV infection and that they may gain a potential benefit from HPV vaccines. In fact, globally there are around 22,000 OPSCC cases annually caused by HPV infection with 80%–90% being due to HPV 16 infection. These cases might be preventable by HPV vaccination (40). Importantly, there has been prospective clinical research to explore the implementation of HPV vaccination in HPV‑associated HNSCC (41). Considerable efforts are needed to further propel HPV vaccination program in HNSCC patients.

As a retrospective study using data from SEER, our study had several limitations. Importantly, due to the nature of the SEER database, information of the HPV test method was not available in the SEER database. Therefore, caution should be taken when interpreting our results about the prevalence of HPV-positive tumors. In addition, data of EBV was incompletely captured in the SEER database, which was a crucial factor for NPC and may lead to a different result for NPC patients. Nonetheless, this study rested on a large sample size to describe the effects of tumor HPV status on HNSCC patients arising from different anatomical subsites including the nasopharynx, oropharynx, and hypopharynx, while prior studies have mainly focused on oropharyngeal cancer.

In conclusion, HPV infection was not low in HNSCC patients, even in non-oropharyngeal HNSCC. HPV status was a crucial clinically applicable prognostic marker in non-nasopharyngeal HNSCC, which suggested that HPV testing was recommended for non-nasopharyngeal HNSCC patients. Prognostic nomograms for OPC and HPC patients including the HPV status were essential for a correct prognosis, and risk classification systems was built which could perfectly classify OPC and HPC patients into low-, intermediated-, and high-risk groups.

## Data Availability Statement

The raw data supporting the conclusions of this article will be made available by the authors, without undue reservation.

## Ethics Statement

Ethical review and approval was not required for the study on human participants in accordance with the local legislation and institutional requirements. The ethics committee waived the requirement of written informed consent for participation.

## Author Contributions

MW: Data collection and data analysis, and writing of the manuscript. QW: Design and data analysis, writing of the manuscript and funding support. YQ, XH, YLiu, and YLi: Data analysis and data collection. XW: data analysis and writing of the manuscript. WL: Data collection and data analysis. YW and YZ: Design, administrative support, resources, and critical revision of the manuscript, funding support. All authors contributed to the article and approved the submitted version.

## Funding

This study was supported by a grant from the Leading Discipline Construction Project of Oncology of Zhongnan Hospital of Wuhan University; a grant from the Science, Technology and Innovation Seed Fund of Zhongnan Hospital of Wuhan University (grant no. znpy2018123); a grant from the Scientific Research Project of Hubei Provincial Health and Family Planning Commission (grant no. WJ2019H064); and a grant from the Science and Technology Innovation and Cultivation program of Zhongnan Hospital of Wuhan University (grant no. znpy 2018114).

## Conflict of Interest

The authors declare that the research was conducted in the absence of any commercial or financial relationships that could be construed as a potential conflict of interest.

## Publisher’s Note

All claims expressed in this article are solely those of the authors and do not necessarily represent those of their affiliated organizations, or those of the publisher, the editors and the reviewers. Any product that may be evaluated in this article, or claim that may be made by its manufacturer, is not guaranteed or endorsed by the publisher.

## Supplementary Material

Supplementary Figure 1 ROC curves depicting predictive performance of the survival nomograms in the validation cohorts. **(A, B)** ROC curves for 3-year and 5-year OS of OPC patients in the validation cohort; **(C, D)** ROC curves for 3-year and 5-year OS of HPC patients in the validation cohort. ROC: receiver-operating characteristic; OS: overall survival; FP: false positive; TP: true positive; OPC: oropharyngeal carcinoma; HPC: hypopharyngeal carcinoma.

Supplementary Figure 2 The calibration curves for predicting OS of OPC and HPC patients in the validation cohorts. **(A, B)** Calibration curves for 3-year and 5-year OS of OPC patients in the validation cohort; **(C, D)** calibration curves for 3-year and 5-year OS of HPC patients in the validation cohort. OS: overall survival; OPC: oropharyngeal carcinoma; HPC: hypopharyngeal carcinoma.
