# Supplementary figures and images for: HPV Positive Status Is a Favorable Prognostic Factor in Non-Nasopharyngeal Head and Neck Squamous Cell Carcinoma Patients: A Retrospective Study From the Surveillance, Epidemiology, and End Results Database
Source: Front Oncol. 2021 Sep 24;11:688615. doi: 10.3389/fonc.2021.688615 (PMC8497986; doi:10.3389/fonc.2021.688615)

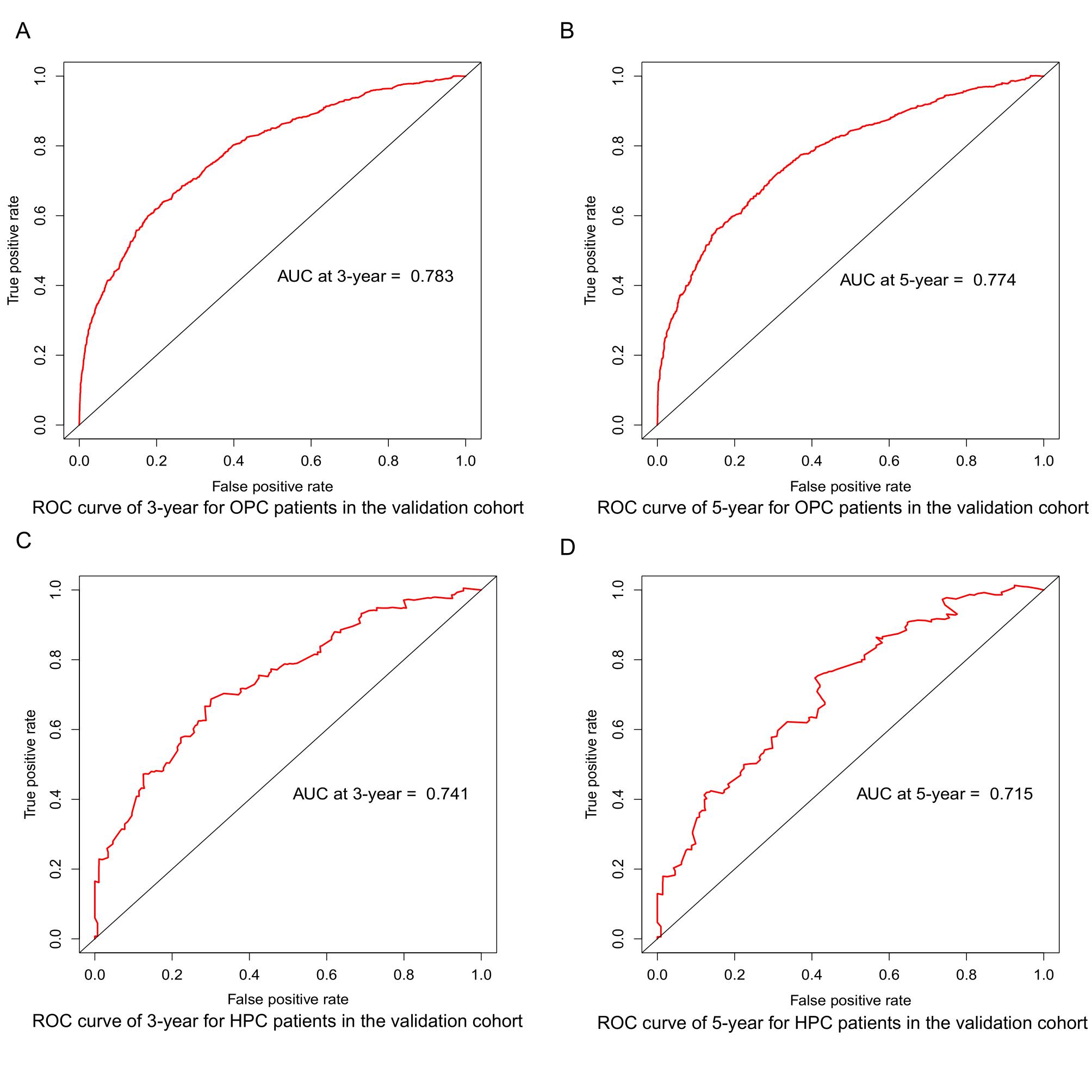

Supplement: Supplementary Figure 1 — ROC curves depicting predictive performance of the survival nomograms in the validation cohorts. (A, B) ROC curves for 3-year and 5-year OS of OPC patients in the validation cohort; (C, D) ROC curves for 3-year and 5-year OS of HPC patients in the validation cohort. ROC: receiver-operating characteristic; OS: overall survival; FP: false positive; TP: true positive; OPC: oropharyngeal carcinoma; HPC: hypopharyngeal carcinoma. [file Image_1.jpeg]

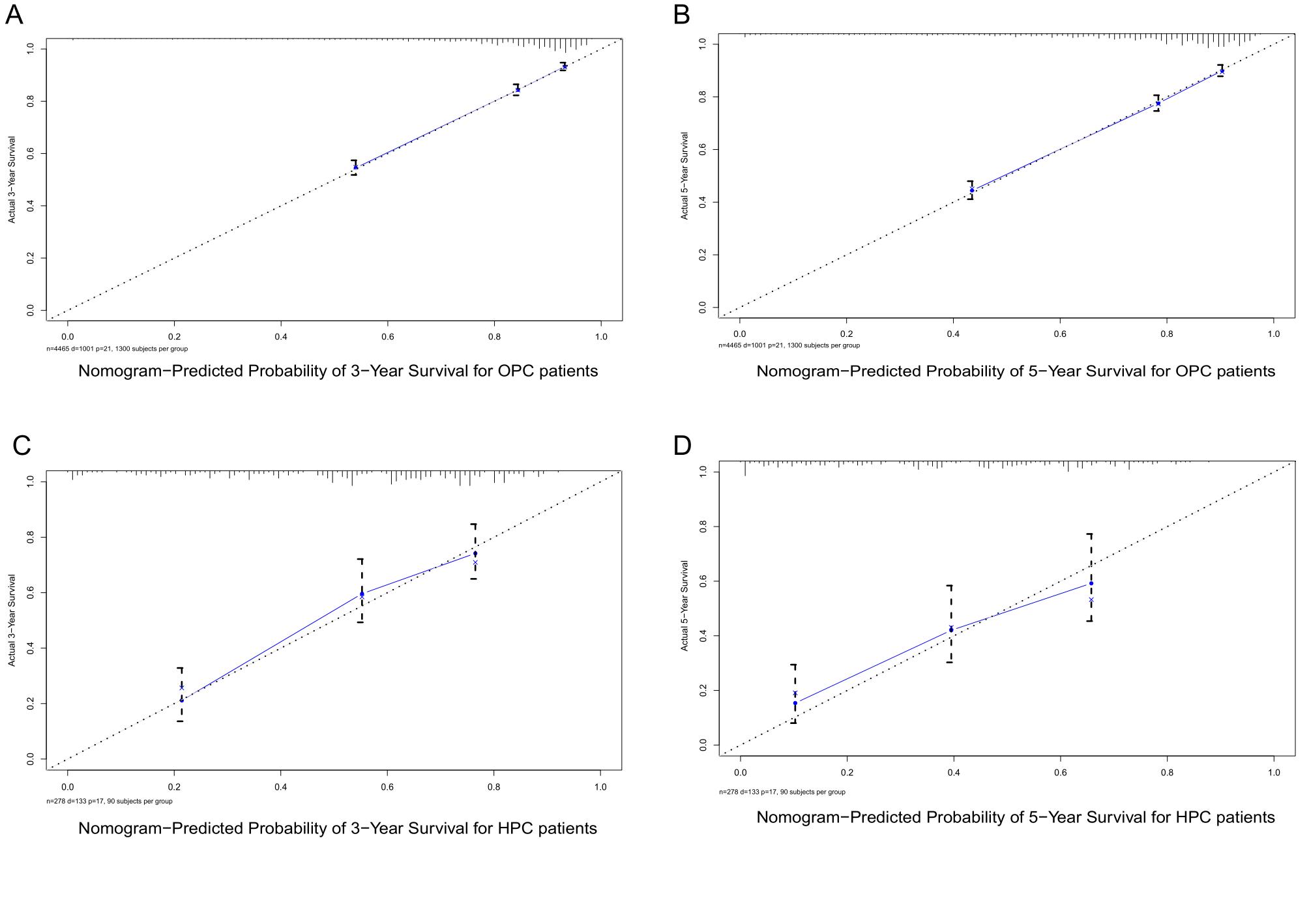

Supplement: Supplementary Figure 2 — The calibration curves for predicting OS of OPC and HPC patients in the validation cohorts. (A, B) Calibration curves for 3-year and 5-year OS of OPC patients in the validation cohort; (C, D) calibration curves for 3-year and 5-year OS of HPC patients in the validation cohort. OS: overall survival; OPC: oropharyngeal carcinoma; HPC: hypopharyngeal carcinoma. [file Image_2.jpeg]
