# Supplementary material for: HPV Positive Status Is a Favorable Prognostic Factor in Non-Nasopharyngeal Head and Neck Squamous Cell Carcinoma Patients: A Retrospective Study From the Surveillance, Epidemiology, and End Results Database
Source: Front Oncol. 2021 Sep 24;11:688615. doi: 10.3389/fonc.2021.688615 (PMC8497986; doi:10.3389/fonc.2021.688615)
Supplement: Supplementary file 3 [file Table_1.docx]

**Supplementary Table 1 Univariate and multivariate cox analysis of overall survival in overall HNSCC patients**

| **Covariate** | **Univariate analysis** | | **Multivariable analysis** | |
| --- | --- | --- | --- | --- |
|  | **HR (95% CI)** | **P-value** | **HR (95% CI)** | **P-value** |
| **HPV status** |  |  |  |  |
| HPV (-) | - | - | - | - |
| HPV (+) | 0.36 (0.33-0.39) | **<0.001** | 0.51 (0.46 - 0.55) | **<0.001** |
| **Primary site** |  |  |  |  |
| Nasopharynx | - | - | - | - |
| Hypopharynx | 1.84 (1.50-2.27) | **<0.001** | 1.28 (1.03 - 1.60) | **0.026** |
| Oropharynx | 0.71 (0.60-0.85) | **<0.001** | 0.97 (0.80 - 1.18) | 0.792 |
| **Age** |  |  |  |  |
| 18-49 | - | - | - | - |
| 50-69 | 1.39 (1.21-1.60) | **<0.001** | 1.37 (1.19 - 1.58) | **<0.001** |
| >=70 | 3.17 (2.73-3.69) | **<0.001** | 2.57 (2.20 - 3.01) | **<0.001** |
| **Race** |  |  |  |  |
| Black | - | - | - | - |
| White | 0.45 (0.40-0.51) | **<0.001** | 0.77 (0.69 - 0.87) | **<0.001** |
| Other ^#^ | 0.57 (0.46-0.69) | **<0.001** | 0.78 (0.63 - 0.96) | **0.020** |
| Unknown | 0.18 (0.06-0.57) | **0.003** | 0.38 (0.12 - 1.20) | **0.010** |
| **Gender** |  |  |  |  |
| Male | - | - | - | - |
| Female | 1.32 (1.19-1.46) | **<0.001** | 1.10 (0.99 - 1.22) | 0.072 |
| **Marital status** |  |  |  |  |
| Married | - | - | - | - |
| Non-married | 2.16 (1.99-2.35) | **<0.001** | 1.63 (1.50 - 1.78) | **<0.001** |
| Unknown | .63 (1.35-1.96) | **<0.001** | 1.27 (1.05 - 1.53) | **0.012** |
| **Grade** |  |  |  |  |
| Grade I-II | - | - | - | - |
| Grade III-IV | 0.62 (0.57-0.68) | **<0.001** | 0.71 (0.64 - 0.77) | **<0.001** |
| Unknown | 0.84 (0.76-0.94) | **0.002** | 0.84 (0.75 - 0.94) | **0.002** |
| **T stage** |  |  |  |  |
| T1 | - | - | - | - |
| T2 | 1.58 (1.38-1.80) | **<0.001** | 1.46 (1.27 - 1.67) | **<0.001** |
| T3 | 3.14 (2.74-3.60) | **<0.001** | 2.38 (2.06 - 2.74) | **<0.001** |
| T4 | 5.22 (4.58-5.95) | **<0.001** | 3.51 (3.05 - 4.03) | **<0.001** |
| N **stage** |  |  |  |  |
| N0 | - | - | - | - |
| N1 | 0.96 (0.83-1.10) | 0.530 | 1.31 (1.14 - 1.51) | **0.0002** |
| N2 | 1.01 (0.90-1.13) | 0.861 | 1.50 (1.33 - 1.70) | **<0.001** |
| N3 | 1.85 (1.56-2.20) | **<0.001** | 2.25 (1.88 - 2.68) | **<0.001** |
| M **stage** |  |  |  |  |
| M0 | - | - | - | - |
| M1 | 5.18 (4.53-5.92) | **<0.001** | 2.75 (2.38 - 3.17) | **<0.001** |
| **Surgery for primary site** |  |  |  |  |
| No | - | - | - | - |
| Yes | 0.41 (0.37-0.45) | **<0.001** | 0.53 (0.47 - 0.59) | **<0.001** |
| Unknown | 1.08 (0.35-3.37) | 0.888 | 1.33 (0.43 - 4.15) | 0.621 |
| **Radiotherapy** |  |  |  |  |
| No | - | - | - | - |
| Yes | 0.40 (0.36-0.44) | **<0.001** | 0.44 (0.39 - 0.49) | **<0.001** |
| **Chemotherapy** |  |  |  |  |
| No | - | - | - | - |
| Yes | 0.80 (0.73-0.87) | **<0.001** | 0.67 (0.61 - 0.75) | **<0.001** |

^#^: American Indian/AK Native, Asian/Pacific Islander

HPV, human papillomavirus; HNSCC, head and neck squamous cell carcinomas; AJCC, American Joint Committee on Cancer.
