# Supplementary material for: HPV Positive Status Is a Favorable Prognostic Factor in Non-Nasopharyngeal Head and Neck Squamous Cell Carcinoma Patients: A Retrospective Study From the Surveillance, Epidemiology, and End Results Database
Source: Front Oncol. 2021 Sep 24;11:688615. doi: 10.3389/fonc.2021.688615 (PMC8497986; doi:10.3389/fonc.2021.688615)
Supplement: Supplementary file 4 [file Table_2.docx]

**Supplementary Table 2: Characteristics of patients with oropharyngeal carcinoma (OPC) and**

**Hypopharyngeal carcinoma (HPC) in both training and validation cohorts**

| **Characteristics** | **OPC** | | **P-value** | **HPC** | | **P-value** |
| --- | --- | --- | --- | --- | --- | --- |
|  | **Training cohort**  **n = 4468** | **Validation cohort**  **n = 4465** |  | **Training cohort**  **n = 280** | **Validation cohort**  **n = 278** |  |
| **HPV status** |  |  | 0.554 |  |  | 0.807 |
| HPV (-) | 1189 (26.6%) | 1214 (27.2%) |  | 208 (74.3%) | 210 (75.5%) |  |
| HPV (+) | 3279 (73.4%) | 3251 (72.8%) |  | 72 (25.7%) | 68 (24.5%) |  |
| **Age** |  |  | 0.770 |  |  |  |
| 18-49 | 561 (12.6%) | 578 (12.9%) |  | 22 (7.9%) | 23 (8.3%) |  |
| 50-69 | 3230 (72.3%) | 3229 (72.3%) |  | 185 (66.1%) | 182 (65.5%) |  |
| >=70 | 677 (15.2%) | 658 (14.7%) |  | 73 (26.1%) | 73 (26.3%) |  |
| **Race** |  |  | 0.438 |  |  | 0.306 |
| Black | 355 (7.9%) | 315 (7.1%) |  | 37 (13.2%) | 35 (12.5%) |  |
| White | 3930 (88.0%) | 3970 (88.9%) |  | 215 (76.8%) | 217(78.1%) |  |
| Other^#^ | 165 (3.7%) | 164 (3.7%) |  | 28 (10%) | 26 (9.3%) |  |
| Unknown | 18 (0.4%) | 16 (0.4%) |  |  |  |  |
| **Gender** |  |  | 0.918 |  |  | 1.000 |
| Male | 3766 (84.3%) | 3768 (84.4%) |  | 229 (81.8%) | 228 (82.0%) |  |
| Female | 702 (15.7%) | 697 (15.6%) |  | 51 (18.2%) | 50 (18.0%) |  |
| **Marital status** |  |  | 0.134 |  |  | 0.448 |
| Married | 2618 (58.6%) | 2660 (59.6%) |  | 134 (47.9%) | 120 (43.2%) |  |
| Non-married | 1634 (36.6%) | 1627 (36.4%) |  | 127 (45.4%) | 141 (50.7%%) |  |
| Unknown | 216 (4.8%) | 178 (4.0%) |  | 19 (6.8%) | 17 (6.1%%) |  |
| **Grade** |  |  | 0.455 |  |  | 0.633 |
| Grade I-II | 1480 (33.1%) | 1534 (34.4%) |  | 124 (44.3%) | 112 (40.3%) |  |
| Grade III-IV | 2032 (45.5%) | 2002 (44.8%) |  | 103 (36.8%) | 110 (39.6%) |  |
| Unknown | 956 (21.4%) | 929 (20.8%) |  | 53 (18.9%) | 56 (20.1%) |  |
| **T stage** |  |  | 0.522 |  |  | 0.308 |
| T1 | 1231 (27.6%) | 1195 (26.8%) |  | 28 (10.0%) | 31 (11.2%) |  |
| T2 | 1703 (38.1%) | 1743 (39.0%) |  | 90 (32.1%) | 107 (38.5%) |  |
| T3 | 802 (17.9%) | 829 (18.6%) |  | 81 (28.9%) | 65 (23.4%) |  |
| T4 | 732 (16.4%) | 698 (15.6%) |  | 81 (28.9%) | 75 (27.0%) |  |
| **N stage** |  |  | 0.400 |  |  | 0.527 |
| N0 | 671 (15.0%) | 711 (15.9%) |  | 74 (26.4%) | 67 (24.1%) |  |
| N1 | 806 (18.0%) | 822 (18.4%) |  | 53 (18.9%) | 45 (16.2%) |  |
| N2 | 2789 (62.4%) | 2714 (60.8%) |  | 139 (49.6%) | 146 (52.5%) |  |
| N3 | 202 (4.5%) | 218 (4.9%) |  | 14 (5.0%) | 20 (7.2%) |  |
| **M stage** |  |  | 0.306 |  |  | 0.587 |
| M0 | 4318 (96.6%) | 4333 (97.0%) |  | 263 (93.9%) | 265 (95.3%) |  |
| M1 | 150 (3.4%) | 132 (3.0%) |  | 17 (6.1%) | 13 (4.7%) |  |
| **Surgery for primary site** |  |  | 0.385 |  |  | 0.606 |
| No | 2747 (61.5%) | 2707 (60.6%) |  | 222 (79.3%) | 222 (79.9%) |  |
| Yes | 1719 (38.5%) | 1753 (39.3%) |  | 57 (20.4%) | 56 (20.1%) |  |
| Unknown | 2 (0.0%) | 5 (0.1%) |  | 1 (0.4%) | 0 (0.0%) |  |
| **Radiotherapy** |  |  | 0.547 |  |  | 0.877 |
| No | 556 (12.4%) | 536 (12.0%) |  | 40 (14.3%) | 42 (15.1%) |  |
| Yes | 3912 (87.6%) | 3929 (88.0%) |  | 240 (85.7%) | 236 (84.9%) |  |
| **Chemotherapy** |  |  | 0.107 |  |  | 0.654 |
| No | 1181 (26.4%) | 1249 (28.0%) |  | 62 (22.1%) | 67 (24.1%) |  |
| Yes | 3287 (73.6%) | 3216 (72.0%) |  | 218 (77.9%) | 211 (75.9%) |  |

HPV: human papillomavirus; AJCC, American Joint Committee on Cancer;

#: AmericanIndian/AK Native, Asian/Pacific Islande**r**
